# Non-pharmaceutical intervention levels to reduce the COVID-19 attack ratio among children

**DOI:** 10.1098/rsos.211863

**Published:** 2022-03-16

**Authors:** Jummy David, Nicola Luigi Bragazzi, Francesca Scarabel, Zachary McCarthy, Jianhong Wu

**Affiliations:** ^1^ Fields-CQAM Laboratory of Mathematics for Public Health (MfPH), York University, Toronto, Ontario, Canada; ^2^ Laboratory for Industrial and Applied Mathematics, York University, Toronto, Ontario, Canada; ^3^ Department of Mathematics, The University of Manchester, Manchester, UK; ^4^ Joint UNIversities Pandemic and Epidemiological Research (JUNIPER), UK; ^5^ CDLab - Computational Dynamics Laboratory, Department of Mathematics, Computer Science and Physics, University of Udine, Italy

**Keywords:** COVID-19, attack ratio, public health and social measures, vaccination, age-related heterogeneity, disease control

## Abstract

The attack ratio in a subpopulation is defined as the total number of infections over the total number of individuals in this subpopulation. Using a methodology based on an age-stratified transmission dynamics model, we estimated the attack ratio of COVID-19 among children (individuals 0–11 years) when a large proportion of individuals eligible for vaccination (age 12 and above) are vaccinated to contain the epidemic among this subpopulation, or the *effective* herd immunity (with additional physical distancing measures). We describe the relationship between the attack ratio among children, the time to remove infected individuals from the transmission chain and the children-to-children daily contact rate while considering the increased transmissibility of virus variants (using the Delta variant as an example). We illustrate the generality and applicability of the methodology established by performing an analysis of the attack ratio of COVID-19 among children in the population of Canada and in its province of Ontario. The clinical attack ratio, defined as the number of symptomatic infections over the total population, can be informed from the attack ratio and both can be reduced substantially via a combination of reduced social mixing and rapid testing and isolation of the children.

## 1. Introduction

While the infection by the ‘Severe Acute Respiratory Syndrome-related Coronavirus type 2’ (SARS-CoV-2) has been reported to affect all age groups, including newborns and infants, children, adolescents and young adults [1], reliable and accurate epidemiological estimates of ‘Coronavirus disease 2019’ (COVID-19) among children are particularly challenging to obtain [2]. The precise role of children in transmitting the pathogen has been controversial and a subject of debate [1–3].

A recently published systematic review and meta-analysis [1] has synthesized 29 early studies of surveillance conducted during the first wave of COVID-19. Authors have found that the rate of children test-positive but clinically asymptomatic was 21.1%, whereas the rate of severely or critically symptomatic children was 3.8%. No studies on COVID-19 transmissibility in children could be retrieved, while susceptibility to COVID-19 among children was highly heterogeneous across studies included in the systematic review and meta-analysis. A recent study conducted in Norwegian families [4] has shown that young children can transmit the COVID-19 virus to the same extent as adults. The available evidence is scarce, of poor quality, and sometimes conflicting. However, cohort/population, network and household studies [5–14] and meta-analyses of household surveys [15–17] seem to suggest that COVID-19 infection among children is generally asymptomatic or has a mild course, with ‘classical symptoms’ such as cough, fever, anosmia and ageusia being less frequently reported with respect to non-specific/gastrointestinal ones [18]. However, as stated by Mehta *et al*. [19] and by Hyde [2], lower secondary attack rates in children may be due to the fact that children are less tested and exposed than adults. This seems more plausible than hypothesizing a biological difference in susceptibility. Some studies seem to confirm this reporting bias: Reukers *et al*. [20] have applied a dense sampling approach in 55 Dutch households, with a total of 187 household contacts, computing a secondary attack rate of 35% among children. This rate is lower than the rate computed among adults (51%), but is considerably higher than the rate among children reported in previously published studies. On the other hand, some data seem to point to a shorter shedding period in children compared with adults, with an immunologically different response to the novel coronavirus in terms of antibody kinetics [2,19].

Moreover, previously reported epidemiological trends may not be updated in those settings and scenarios characterized by increased circulation of variants of the COVID-19 virus, such as variant B.1.1.7 (also known as the Alpha variant). A recent study conducted in Germany [21] found that the secondary attack rate among children could be similar to the rate among adults. With children going back to school, it is of paramount importance to computing the impact of reopened schools on COVID-19 transmission dynamics. Sero-epidemiological surveys and contact tracing studies, such as the study conducted by Boey *et al.* [22], are needed, as well as mathematical models to inform and guide the decision-making process of public health decision- and policy-makers in terms of protocols and interventions to adopt and implement.

The present modelling study aimed at estimating the attack ratio among children currently not eligible for vaccination, when vaccination coverage alone (in the vaccine-eligible age group) or together with the implementation of some additional non-pharmaceutical interventions (such as the use of face masks or physical distancing) can significantly reduce the transmission in the vaccine-eligible population. We here develop a methodology, based on analysis of a disease transmission dynamics model, to estimate the age-stratified attack ratio within a subpopulation. We then demonstrate the usage of this methodology by estimating the attack ratio of COVID-19 among children (specifically, individuals 0–11 years of age) in Ontario, Canada, and in the entire country Canada, as a function of different adjustable model parameters, such as their activity levels and time to removal from the transmission chain. We explore the sensitivity of the attack ratio on these key parameters to gain insights into practical recommendations as how to reduce the attack ratio of COVID-19 among children, which is key to ultimately reducing disease burden in this subpopulation, while reopening schools and partially resuming social and economic activities in the entire population.

## 2. Methods

### 2.1. The study setting and transmission dynamics model

We constructed a transmission dynamics model to calculate the attack ratio of COVID-19 among children (0–11 years), defined as the total number of infected children over the total number of children in this age interval. Using the transmission model, we characterized explicitly the attack ratio in each age group from the model parameters. While this methodology is very general, we parametrized the model with Canada and its province of Ontario using publicly available data [23–25]. We then estimated the attack ratio among children against a variety of adjustable model parameters, including the daily contact rate within the vaccine-eligible population (12 years of age and older), the children-to-children daily contact rate (which we used as a proxy for school opening capacity), the testing and isolation of infectious individuals (potentially through contact tracing). In our analysis, we also considered the increased transmissibility of the Delta variant and a decreased transmissibility through the utilization of masks and other physical distancing measures.

In our model, the population is divided into susceptible (*S*), exposed (*E*), asymptomatic infectious (*A*), infectious with symptoms (*I*) and recovered (*R*) compartments according to the epidemiological status of individuals. The population is further stratified by age, sub-index 1 for those eligible for vaccination (12 years and older) while sub-index 2 for children (0–11 years), so, for example, *S*_1_ is the compartment of susceptible individuals eligible for vaccination, while *I*_2_ is the compartment for symptomatically infected children. The transmission dynamics model is given by a system of ordinary differential equations as follows:

$$S_{i}^{\prime }=-\beta_{i}S_{i}\left[\frac{C_{i1}(I_{1}+\theta A_{1})}{N_{1}}+\frac{C_{i2}(I_{2}+\theta A_{2})}{N_{2}}\right],$$

$$E_{i}^{\prime }=\beta_{i}S_{i}\left[\frac{C_{i1}(I_{1}+\theta A_{1})}{N_{1}}+\frac{C_{i2}(I_{2}+\theta A_{2})}{N_{2}}+\right]-\delta_{i}E_{i},$$

$$A_{i}^{\prime }=(1-\rho )\delta_{i}E_{i}\;-\gamma_{i}A_{i}$$

$$\mathrm{and}\;\;\;\;\;\;\;\;\;I_{i}^{\prime }=\rho \delta_{i}E_{i}-\gamma_{i}I_{i}\;,$$

for each age group *i* = 1, 2. The model parameters, with the appropriate sub-indices (1 for vaccine eligible (12 + years), 2 for children (0–11 years), are defined as follows:

 *β*: individual susceptibility, describing the transmission probability per contact;
 *θ*: the relative infectiousness of asymptomatic infectious versus the symptomatic infectious;
 *δ*: the inverse of the latent period;
 *ρ*: the proportion of exposed individuals becoming symptomatic infectious;
 *γ*: the rate at which the infected individuals are removed (via recovery or isolation).

The contact mixing is described by a matrix *C* where each entry *C_ij_* represents the number of daily contacts of one individual in age group *i* with individuals in age group *j*. *N*_1_ and *N*_2_ are the total number of vaccine-eligible individuals and children, and *S*_1_(0) is the initial population of vaccine-eligible susceptible individuals, and this is (given that the number of initially infected vaccine-eligible individuals is small) *S*_1_(0) = (1 − *p*)*N*_1_, with

 *p*: the effective vaccine coverage, defined as the percentage of vaccinated individuals in the vaccine-eligible population multiplied by the effectiveness of vaccination against infection.

### 2.2. The age group-specific attack ratio

By definition, the age group-specific attack ratio among vaccine-eligible individuals and children is given by *a_i_* = (*S_i_*_,0_ − *S_i_*_,∞_)/*N_i_*, the difference of the initial and final size of the susceptible individuals in the population, divided by the total population. Through a mathematical analysis of the transmission dynamics model introduced in §2.1, we obtained a system of nonlinear equations which we solved to estimate the age group-specific attack ratio (see appendix A). Specifically, these were calculated by solving the coupled system of nonlinear algebraic equations (the so-called final size equations) for *x*_1_ = 1 − *a*_1_, *x*_2_ = 1 − *a*_2_,

$$x_{1}=e^{-\beta_{1}^{*}\left[\frac{C_{11}\rho (1-p)}{\gamma_{1}}(1-x_{1})+\frac{C_{12}\rho }{\gamma_{2}}(1-x_{2})\right]}$$

and

$$x_{2}=e^{-\beta_{2}^{*}\left[\frac{C_{21}\rho (1-p)}{\gamma_{1}}(1-x_{1})+\frac{C_{22}\rho }{\gamma_{2}}(1-x_{2})\right]},$$

where $\beta_{1}^{⋆}=\beta_{1}[1+(\theta (1-\rho )/\rho )]$ and $\beta_{2}^{⋆}=\beta_{2}[1+(\theta (1-\rho )/\rho )]$ describe the modified transmission rates that account for symptomatic and asymptomatic transmission.

### 2.3. Contact mixing in Ontario and Canada

Similarly, as done in [26], we calculated the pre-pandemic contact mixing in Ontario and Canada for the year 2020 using the age-specific contact matrix established for the Canadian setting in year 2006 in [27] and the methods used to adjust contact mixing matrices according to different population age structure profiles [28]. We here present the key steps of the method for the Ontario mixing matrix calculation; the contact mixing matrix for Canada is computed similarly.

Denote by *C* the established mixing matrix estimated in Canada from Prem *et al*. [27], which provides the 16-age class matrix in terms of 5-year bands (i.e. 0–4 years, 5–9 years, 10–14 years,…). We corrected the reference matrix *C* to ensure that the reciprocity condition is satisfied (*C_ij_N_i_* = *C_ji_N_j_* for all *i*, *j*) by applying the matrix transformation

$$C_{ij\;}\to \frac{1}{2N_{i}}(C_{ij}N_{i}+{\left(C_{\;ji}N_{j}\right)}^{T}),$$

which balanced the population-level contacts between age classes through making the extensive scale matrix symmetric. Next, the reference matrix for Canada was adjusted to the Ontario setting considering age structure profiles in Canada and Ontario in 2006 using an established method (Method M3 in [28]). We then calculated, from the 16-age class mixing matrix *C*, the mixing matrix in terms of the two age classes of the present study (0–11, 12 + years), using a transformation based on the homogeneous mixing assumption. The entries of the 2 × 2 matrix were computed using

$$C_{kl\;}^{2}=\frac{1}{N_{k}^{2}}\overset{16}{\underset{i=1}{\sum }}\overset{16}{\underset{j=1}{\sum }}C_{ij}N_{i}\frac{{\bar{N}}_{\;jl}\;}{N_{j}}\frac{{\bar{N}}_{ik}}{N_{i}},\;$$

where ${\bar{N}}_{jl}$ (${\bar{N}}_{ik}$) represents the overlapped population in the original age group *j* (*i*) and present study age group *l* (*k*). Also, *C_ij_* and $C_{kl}^{2}$ are the entries of the contact matrix for the old and new age structure in Ontario, respectively. *N_i_* is the population of age group *i* (*i* = 1, … ,16) for the 2006 age distribution in Ontario, while $N_{k}^{2}$ is the population in age group *k* of the new two-group division (*k* = 1, 2) in 2006 in Ontario. We applied a final correction to adjust the contact matrix $C_{kl}^{2}$ to that of the year 2020. To do this, we used the density transformation (Method M2 in [28]) using population data for 2006 and 2020 in Ontario to update appropriately.

We obtained the Ontario contact mixing matrix entries:

$$C_{11}\;=\;12.73,\;C_{12}\;=\;1.03,\;C_{21}\;=\;7.39,\;C_{22}\;=\;4.3,$$

resulting in a mean connectivity of 13.5 daily contacts. The population profile for Ontario for the two age groups are *N*_1_ = 12 932 471, *N*_2_ = 1 801 543 [25].

Similarly, we have for Canada the following:

$$C_{11}\;=\;11.27,\;C_{12}\;=\;0.99,\;\;C_{21}\;=\;6.85,\;\;C_{22}\;=\;4.47,$$

resulting in a mean connectivity of 12.14 daily contacts. The population profile for Canada for the two age groups are *N*_1_ = 33 198 268, *N*_2_ = 4 806 970 [25].

### 2.4. Effective vaccination coverage

The vaccine effectiveness against all infections by variant of concern (VoC) Delta for a single dose and two doses of BNT162b2 was estimated to be 57% and 80%, respectively [29]. We computed the effective vaccine coverage *p* by considering the protection granted by both the partial (one dose) and full vaccination (two doses) among vaccine-eligible individuals in the Ontario population as

 3% of coverage among vaccine-eligible [23] times 57% effectiveness against infection for single-dose status = 0.03 × 0.57, plus
 86% of coverage among vaccine-eligible [23] times 80% of effectiveness against infection for two-dose status = 0.86 × 0.80,

which yields a total of 70.5% effective coverage for both vaccination statuses, as of 19 November 2021.

For Canada, we computed the effective vaccine coverage *p* in a similar way, as:

 3.50% of coverage among vaccine-eligible [24] times 57% of effectiveness [29] against infection for a single-dose status = 0.035 × 0.57, plus
 85.49% of coverage [24] times 79% of effectiveness [29] against infection for a two-dose status = 0.8549 × 0.80,

which gives about 70.39% of effective coverage for all vaccinations, as of 19 November 2021.

### 2.5. Non-VoC and VoC (the Delta variant) transmission rate

We estimated the baseline value for the modified transmission rate $\beta_{1}^{*}$ for the ancestral (non-VoC) strain from pre-pandemic values by inverting the formula

$$R_{1,0}=\beta_{1}^{*}\frac{C_{11}}{\gamma_{1}}.$$

This formula is obtained under the assumption that, at the beginning of the epidemic, all contribution to the transmission was due to contacts within the vaccine-eligible group, with the contribution of children to transmission being negligible. Hence, *R*_1,0_ can be taken approximately as the reproduction number during the first COVID-19 epidemic wave: we assumed a baseline reproduction number *R*_1,0_ = 2.5 [30]. Furthermore, we assumed a removal rate *γ*_1_ = 1/7 without interventions (hence, only due to recovery) [31] and *ρ* = *ρ*_1_ = *ρ*_2_ = 0.7 [32].

For the province of Ontario, using these parameters and pre-pandemic contact rates (*c*_11_ = 12.73), we estimated $\beta_{1}^{*}=0.0281$ for the ancestral (non-VoC) strain. For Canada, using these parameters and pre-pandemic contact rates (*c*_11_ = 11.27), similar calculations and assumptions led to the estimate $\beta_{1}^{*}=0.0317$ for the ancestral (non-VoC) strain.

To obtain the transmission parameters for the Delta variant, we assumed an increase in the transmissibility from the non-VoC to the Alpha variant by 40% [33,34] and an increased transmissibility from the Alpha to the Delta variant by 60% [35,36], giving $\beta_{1}^{*}=0.0628$ for the Delta variant in Ontario, and $\beta_{1}^{*}=0.0710$ for the Delta variant in Canada.

The modified transmissibility of children, $\beta_{2}^{*}$, for both Ontario and Canada was computed by making assumptions on the relative susceptibility of children compared with adults. Due to the uncertainty in this parameter, we conducted a sensitivity analysis as explained in the next section.

### 2.6. Simulation strategy and scenario analysis

We assessed the attack ratio in children according to different scenarios of the activity levels *C*_11_, *C*_22_, the time to removal 1/*γ*_1_, 1/*γ*_2_ (5, 6, 7 days), and the probability of transmission per contact for children aged 0–11 years of age varying the relative susceptibility of children compared with adults ($\beta_{2}^{*}/\beta_{1}^{*}$) by 40%, 50%, 60% and 70% [37,38], with $\beta_{1}^{*}$ computed in the previous section, resulting in $\beta_{2}^{*}=0.0251,0.0314,0.0377,\;0.044$, respectively, for the province of Ontario, and $\beta_{2}^{*}=0.0284,0.0355,0.0426,0.0497,$ respectively for the entire country of Canada. With these parameters and the other parameters defined in §§2.3–2.5, we estimated the attack ratio in children as explained in §2.2.

## 3. Results

### 3.1. Attack ratio among children in Ontario and impact of public health measures

We investigated a variety of scenarios to assess the impact of several adjustable model parameters on the attack ratio of COVID-19 among children 0–11 years of age in Ontario. Table 1 summarizes the numerical results by assuming that the relative susceptibility of children compared with adults ($\beta_{2}^{*}/\beta_{1}^{*}$) is 50% (i.e. $\beta_{2}^{*}=\;0.5\times \beta_{1}^{*}=0.0314$), for different removal rates when the activity among the vaccine-eligible population reaches its pre-pandemic level (*C*_11_ = 12.73), and the Stage 3 reopening level (*C*_11_ = 10) estimated in Ontario [39]. We considered the value of *γ*_1_ = *γ*_2_ as 1/7, 1/6 and 1/5, so infected individuals were assumed to be removed from the transmission chain (either through isolation or recovery) after 7, 6 and 5 days on average. These values represent approximately natural recovery without additional isolation measures (7 days) or a reduced infectious period due to some isolation effort (5 and 6 days). Furthermore, we varied the value of the children-to-children daily contact rate (*C*_22_), used here as a proxy for school opening capacity.

**Table 1.** Attack ratio among children 0–11 years of age in Ontario, assuming the relative susceptibility of children compared with adults ($\beta_{2}^{*}/\beta_{1}^{*}$) is 50% (i.e. $\beta_{2}^{*}=\;0.5\times \beta_{1}^{*}=0.0314$).

children-to-children contact rate	days prior to isolation	attack ratio among children (%)
*when the activity of vaccine-eligible population reaches its estimated pre-pandemic level* (*C*_11_ = 12.73)		
4.3	7	31.58
2.15	7	19.12
0	7	12.62
4.3	6	15.18
2.15	6	7.50
0	6	4.27
4.3	5	0.059
2.15	5	0.025
0	5	0.016
*when the activity of vaccine-eligible population reaches the Stage 3 reopening level* (*C*_11_ = 10)		
4.3	7	20.43
2.15	7	6.78
0	7	1.93
4.3	6	0.20
2.15	6	0.03
0	6	0.016

When *γ*_1_ = *γ*_2_ = 1/7 and *C*_11_ = 12.73 (baseline scenario, no interventions in place), and considering the current vaccine uptake (70.5% effective vaccine coverage, see §2.4), the results are alarming; the attack ratio was estimated to reach 31.58% with full children-to-children activity levels (*C*_22_ = 4.3) and decreased to 19.12% and 12.62% when these children-to-children contacts are reduced by half (*C*_22_ = 2.15) or eliminated completely (*C*_22_ = 0).

An increased isolation effort in both age groups (*γ*_1_ = *γ*_2_ = 1/5) allows to contain the epidemic among children even when the contact rates among the vaccine-eligible population are at pre-pandemic levels (*C*_11_ = 12.73): in this case, the estimated attack ratios remain well below 0.1%.

Under the current vaccine uptake and a removal rate of 1/6 (average infectious period of 6 days), to avoid an outbreak sustained by the vaccine-eligible population, the activity level of the vaccine-eligible population must be below 10 contacts per day (table 1).

**Figure 1.**
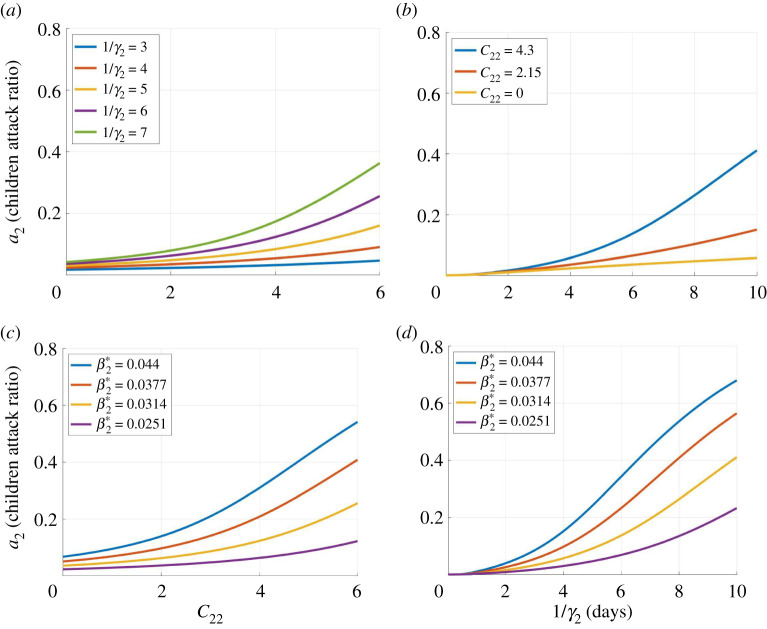
Attack ratio of COVID-19 among children ages 0–11 years in Ontario. The attack ratio is depicted as a function of the children-to-children contact rate *C*_22_ (*a*,*c*) and of the children removal rate $1/\gamma_{2}$ (*b*,*d*). We have considered different values of $1/\gamma_{2}$ (row 1, (*a*)), *C*_22_ (row 1, (*b*)), and $\beta_{2}^{*}$ (row 2) when $1/\gamma_{1}=6$ and *C*_11_ = 12.73.

**Figure 2.**
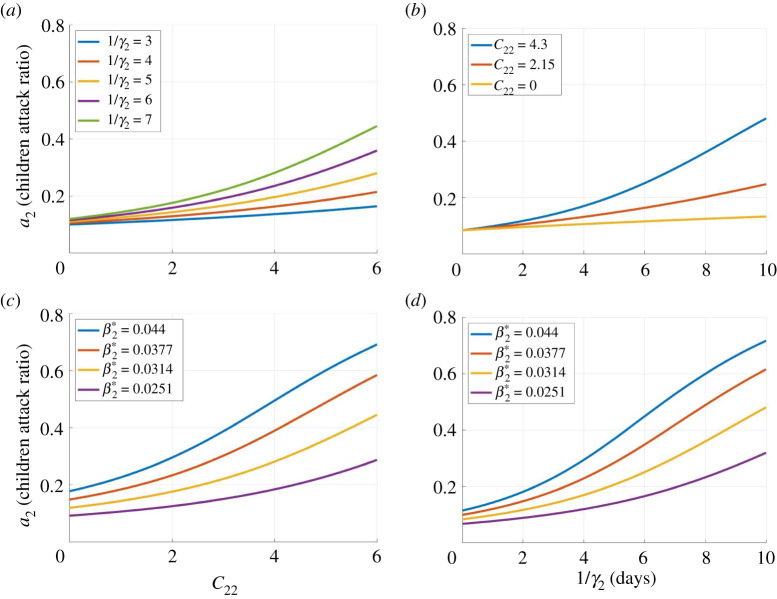
Attack ratio of COVID-19 among children ages 0–11 years in Ontario. The attack ratio depicted as a function of *C*_22_ (*a*,*c*) and (*b*,*d*) for different values of $1/\gamma_{2}$ (row 1, (*a*)), *C*_22_ (row 1, (*c*)) and $\beta_{2}^{*}$ (row 2) when 1/*γ*_1_ = 7 and *C*_11_ = 12.73.

**Figure 3.**
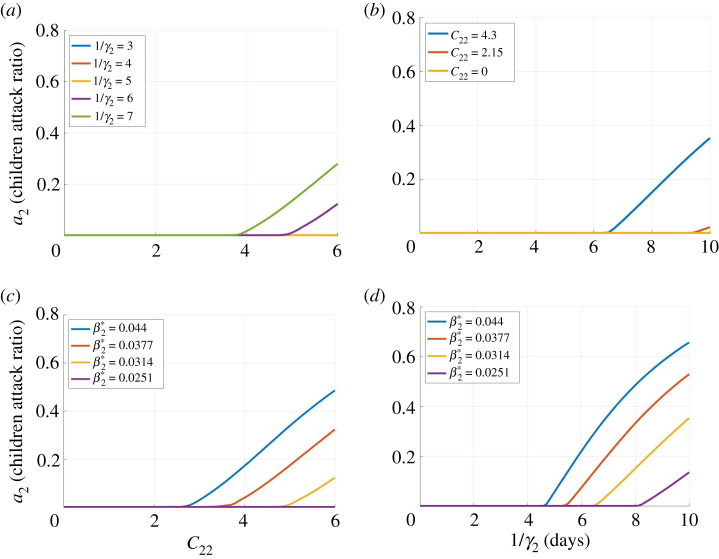
Attack ratio of COVID-19 among children ages 0–11 years in Ontario. The attack ratio is assessed as a function of *C*_22_ (*a*,*c*) and $1/\gamma_{2}$ (*b*,*d*) for different values of $1/\gamma_{2}$ (row 1, (*a*)), *C*_22_ (row 1, (*c*)) and $\beta_{2}^{*}$ (row 2) when 1/*γ*_1_ = 6 and *C*_11_ = 10.

Figures 1–3 in appendix B provide additional illustrations of the effect of important model parameters on the attack ratio in children, *a*_2_, in the cases when the contact levels and time to removal from the transmission chain in the vaccine-eligible population are large enough to drive the epidemic. We explored in particular the effects of the children-to-children contact rate (*C*_22_), the time to removal of infectious children from the transmission chain (1/*γ*_2_) and the children transmission probability per contact ($\beta_{2}^{*}$), obtained by varying the relative susceptibility of children versus the vaccine-eligible population (i.e. varying the ratio $\beta_{2}^{*}/\beta_{1}^{*}$).

Figure 1 considers the case when *C*_11_ = 12.73 and 1/*γ*_1_ = 6, representing a situation where the contact rate of vaccine-eligible individuals is at the pre-pandemic level, but public health efforts reduce the infectious time to 6 days on average. Specifically, the attack ratio *a*_2_ was explored with respect to different children-to-children daily contact rates, different values of the children's transmission probability per contact, and different values of the time to removal of infectious children from the transmission chain. Note that all these parameters have substantial effects on the final attack ratio.

In figure 2, we explored the impact of a delay in the removal of vaccine-eligible infectious individuals (1/*γ*_1_ = 7) from the transmission chain on the attack ratio among children 0–11 years. In other words, in figure 2, we also considered a pre-pandemic contact rate in the vaccine-eligible population (*C*_11_ = 12.73), but assuming it takes 7 days rather than 6 days (as in figure 1) for an infectious individual to be removed from the transmission chain, for different values of *C*_22_, 1/*γ*_2_ and $\beta_{2}^{*}$. It is evident that a delay in the removal rate causes an increase in the attack ratio, which can be quantified for specific choices of parameters.

Figure 3 explores the effect of two combined public health measures, with a removal rate of vaccine-eligible individual of 6 days on average (1/*γ*_1_ = 6) and additional social distancing to keep the contact rate among vaccine-eligible individuals to the Stage 3 reopening levels (*C*_11_ = 10). Again, we explored the dependence of the attack ratio in children on *C*_22_, $\beta_{2}^{*}$ and 1/*γ*_2_.

Overall, the attack ratio is highly sensitive to changes in the transmission probability per contact among children aged 0–11 years old. Specifically, from figures 1, 2 and 3, when the activity of vaccine-eligible population reaches its estimated pre-pandemic (*C*_11_ = 12.73) or the Stage 3 reopening level (*C*_11_ = 10), the attack ratio increases for different values of *C*_22_ and 1/*γ*_1_ as we vary $\beta_{2}^{*}$ from 0.0251 to 0.0314, 0.0377, 0.044.

### 3.2. Attack ratio among children in Canada and impact of public health measures

As a second case study, we performed similar analyses for the entire country of Canada. Table 2 summarizes the results by assuming the relative susceptibility of children compared with adults ($\beta_{2}^{*}/\beta_{1}^{*}$) is 50% (i.e. $\beta_{2}^{*}=\;0.5\times \beta_{1}^{*}=0.0355$), when the activity of the vaccine-eligible population reaches its pre-pandemic level (*C*_11_ = 11.27) or the reduced contact level estimated in the Stage 3 reopening in Ontario (*C*_11_ = 10). We considered the value of *γ*_1_ = *γ*_2_ as 1/5, 1/6, 1/7, so infected individuals can be removed from the transmission chain (either through isolation or recovery) after 5, 6 and 7 days on average, and varied the value of the children-to-children daily contacts per day. The effective vaccine coverage was taken as 70.39% (see §2.4).

**Table 2.** Attack ratio among children 0–11 years of age in Canada, assuming that the relative susceptibility of children compared with adults ($\beta_{2}^{*}/\beta_{1}^{*}$) is 50% (i.e. $\beta_{2}^{*}=\;0.5\times \beta_{1}^{*}=0.0355$).

children-to-children contact rate	days prior to isolation	attack ratio among children (%)
*when the activity of vaccine-eligible population reaches its estimated pre-pandemic level (C*_11_ = 11.27)		
4.47	7	39.43
2.235	7	22.40
0	7	13.73
4.47	6	21.85
2.235	6	9.77
0	6	5.08
4.47	5	0.096
2.235	5	0.013
0	5	0.0072
*when the activity of vaccine-eligible population reaches the Stage 3 reopening level (C*_11_ = 10)		
4.47	7	35.36
2.235	7	17.11
0	7	9.01
4.47	6	15.08
2.235	6	2.56
0	6	0.051
4.47	5	0.015
2.235	5	0.0062
0	5	0.0038

For a pre-pandemic mixing in the vaccine-eligible population (*C*_11_ = 11.27), the results are similar to what is observed for Ontario (table 2). For the baseline removal rate *γ*_1_ = *γ*_2_ = 1/7, representing the natural recovery rate without additional isolation measures, the attack ratio was estimated to reach 39.43% with full children-to-children activity levels (*C*_22_ = 4.47) and decreased to 22.40% and 13.73% when these contacts are reduced by half (*C*_22_ = 2.235) or eliminated completely (*C*_22_ = 0). Under these contact mixing patterns, only a removal rate of about *γ*_1_ = *γ*_2_ = 1/5 allows to contain the epidemic among children (table 2).

With a reduced contact rate of vaccine-eligible population of 10 contacts per day and the current vaccination level (70.39% effective coverage), the situation appears to be worse than what was observed for Ontario: in this case, a removal rate of 1/6 is not sufficient to avoid an outbreak in the children population, unless additional measures are in place to reduce the children-to-children contact in schools from the pre-pandemic level (*C*_22_ = 4.47) to very low levels. A further intensification of isolation of infected individuals (to less than 5 days) becomes necessary in order to maintain pre-pandemic school activities (table 2).

**Figure 4.**
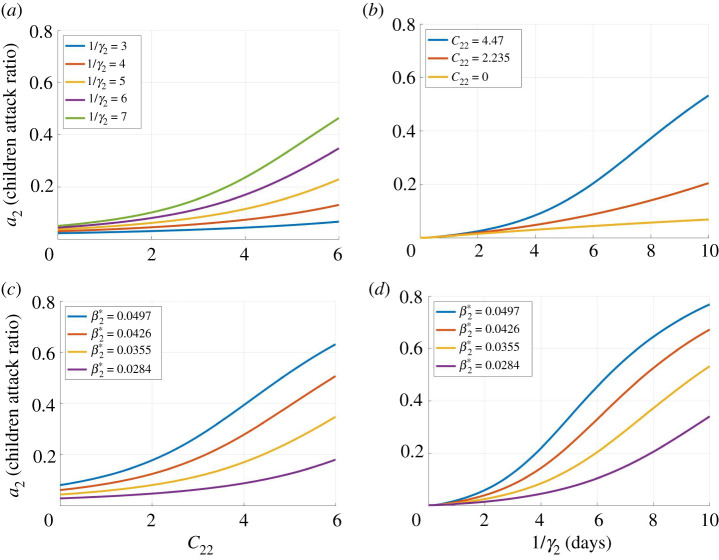
Attack ratio of COVID-19 among children ages 0–11 years in Canada. The attack ratio is depicted as a function of *C*_22_ (*a*,*c*) and $1/\gamma_{2}$ (*b*,*d*) for different values of $1/\gamma_{2}$ (row 1, (*a*)), *C*_22_ (row 1, (*c*)), and $\beta_{2}^{*}$ (row 2) when $1/\gamma_{1}=6$ and *C*_11_ = 11.27.

**Figure 5.**
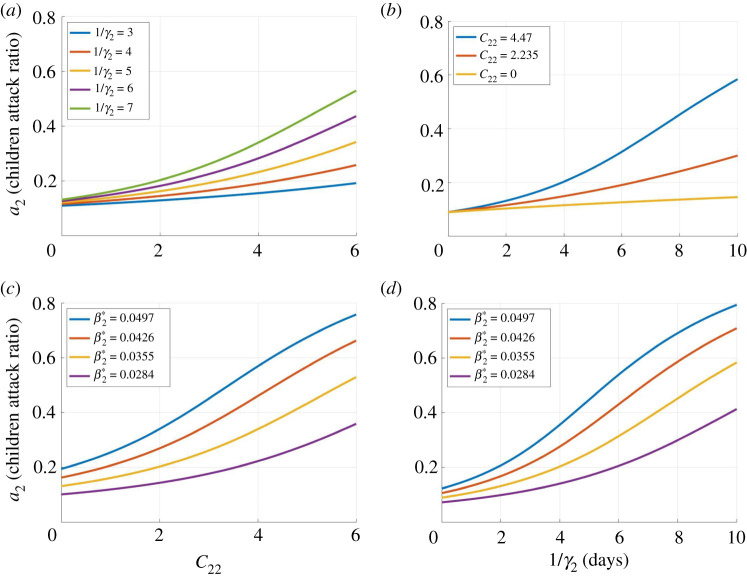
Attack ratio of COVID-19 among children ages 0–11 years in Canada. The attack ratio depicted as a function of *C*_22_ (*a*,*c*) and $1/\gamma_{2}$ (*b*,*d*) for different values of $1/\gamma_{2}$ (row 1, (*a*)), *C*_22_ (row 1, (*c*)) and $\beta_{2}^{*}$ (row 2) when 1/*γ*_1_ = 7 and *C*_11_ = 11.27.

**Figure 6.**
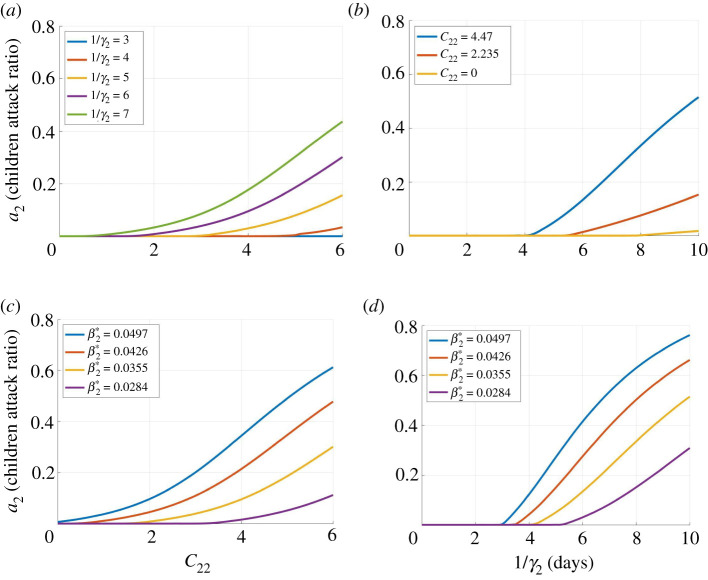
Attack ratio of COVID-19 among children ages 0–11 years in Canada. The attack ratio is assessed as a function of *C*_22_ (*a*,*c*) and $1/\gamma_{2}$ (*b*,*d*) for different values of $1/\gamma_{2}$ (row 1, (*a*)), *C*_22_ (row 1, (*c*)) and $\beta_{2}^{*}$ (row 2) when 1/*γ*_1_ = 6 and *C*_11_ = 10.

Figures 4–6 in appendix B provide additional analyses on the effect of important parameters on the children attack ratio *a*_2_, particularly in regard to the sensitivity to different children-to-children daily contact rates, *C*_22_, different values of the children transmission probability per contact $\beta_{2}^{*}$ and different values of the time to removal from the transmission chain 1/*γ*_2_.

Figure 4 considers the case of a pre-pandemic contact rate (*C*_11_ = 11.27) and removal of vaccine-eligible individuals after 6 days on average (1/*γ*_1_ = 6). Similarly as for the case of Ontario, the analyses show the high sensitivity of the attack ratio to the model parameters.

Figure 5 illustrates the impact of a delay in the removal of infectious individuals from the transmission chain on the attack ratio among children 0–11 years, for pre-pandemic contact rates (*C*_11_ = 11.27) and assuming it takes 7 days (rather than the 6 days in figure 4) for an infectious individual to be removed from the transmission chain. This figure emphasizes an increase in the attack ratio due to a delay in the removal of infectious individuals.

In figure 6, we investigated the case of reduced contacts among vaccine-eligible individuals (*C*_11_ = 10) assuming 6 days to remove individuals from the system (1/*γ*_1_ = 6). Compared with the case of Ontario (figure 3), it is evident that an outbreak among children is expected for most parameter values, under pre-pandemic school mixing (*C*_22_ = 4.47). However, the value of the attack ratio strongly depends on the exact values of the parameters.

Similar to the province of Ontario, the attack ratio in Canada is highly sensitive to changes in the transmission probability per contact among children aged 0–11 years old. Specifically, from figures 4–6, when the activity of vaccine-eligible population reaches its estimated pre-pandemic (*C*_11_ = 11.27) or the Stage 3 reopening level (*C*_11_ = 10), the attack ratio increases for different values of *C*_22_ and $1/\gamma_{1}$ as we vary $\beta_{2}^{*}$ from 0.0284 to 0.0355, 0.0426, 0.0497.

## 4. Discussion and conclusion

Investigating the epidemiology of COVID-19 among children is of crucial importance to inform decisions on how to devise and implement interventions aimed at preserving educational continuity and minimizing the disruption induced by the virus as much as possible.

In the existing literature, some mathematical models have been used to simulate the effects of reducing children-to-children contacts. Abdollahi *et al*. [40] devised an age-structured agent-based simulation model to simulate the effects of closing schools in Ontario, Canada, on the COVID-19 epidemic curve. The authors found that, without forcing self-isolation of mild symptomatic cases, the impact was very limited, in terms of reduced intensive care unit admissions. The burden imposed by the coronavirus was significantly reduced by the implementation of self-isolation practice. However, the precise effect of shutting/reopening schools may depend on the specific setting/country [41]: in countries/territories where community transmission is low, school closing is a non-pharmaceutical intervention characterized by a limited impact, whereas it becomes more relevant in countries where community transmission is higher and more sustained. Some outbreaks have been, indeed, reportedly linked to school communities, for example, in Israel [42], even though other observational studies could not find additional health risks generated by school reopening and in-person attendance [43–46]. All the aforementioned mathematical models and their analyses led to recommendation of continuing implementation of non-pharmaceutical interventions, such as self-isolation and robust test-and-trace measures. This recommendation is particularly valid in those settings and scenarios characterized by an increasing circulation of variants of the COVID-19 virus, which have demonstrated increased transmissibility. In these settings, the computed secondary attack rate among children is not dissimilar to the rate among adults [21].

In the present study, we have devised an algorithm to calculate the attack ratio among children under different scenarios of the activity level of the vaccine-eligible population and the children population, as well as the speed at which infectious individuals are removed from the transmission chain. One important advantage of our approach is that the attack ratio can be described by an explicit and transparent equation, making it possible and efficient to investigate the impact of the parameter values on the population outcome, and understand what interventions on the parameters may allow to reduce the epidemic burden.

We remark that our algorithm is generic and therefore the attack ratio analysis presented within may be conducted for different geographic regions: we have here demonstrated its application to the Canadian province of Ontario and to the entire country of Canada. With the increased transmissibility of the Delta variant, assuming the children are half as susceptible as adults, pre-pandemic contact mixing among vaccine-eligible individuals leads to substantial outbreaks among the children group despite the current levels of vaccination (effective vaccine coverage taken as 70.39% among vaccine-eligible), unless intensified isolation measures reduce the time to removal from the transmission chain to less than 5 days on average. Assuming that infectious individuals can be isolated within 6 days on average from the onset of infectiousness (e.g. by isolation following testing and tracing), a reduction from 12.73 to 10 contacts per day within the vaccine-eligible population in Ontario is necessary to avoid an outbreak among children sustained by the vaccine-eligible population. These values were calculated using a vaccine uptake as of November 2021, and with the currently known values of vaccine efficacy against the Delta variant. We remark that 10 contacts per day correspond to the social mixing level estimated during the Stage 3 reopening in Ontario [39].

The attack ratio among children depends critically on how quickly infectious individuals are removed from the transmission chain: for instance, assuming 10 contacts per day within the vaccine-eligible population, a removal time increasing from 6 to 7 days means that the attack ratio among children with full school activity level is estimated to increase from no outbreak (for a 6-day removal) to an outbreak with attack ratio of 20.43% (for a 7-day removal).

Similar patterns have been observed for Canada: under the current vaccination uptake (70.39% effective coverage) and assuming that children are half as susceptible as adults, maintaining pre-pandemic contact mixing in the vaccine-eligible population would lead to a substantial outbreak among children, unless intense public health measures are in place that reduce the time to isolation of infectious individuals to less than 5 days. Our estimates show that the time to removal cannot be increased even if the contact rate in the vaccine-eligible population is reduced to 10 contacts per day. In this case, removing infected individuals in less than 5 days is necessary to allow schools to reopen to full pre-pandemic activity.

Due to the uncertainty in the estimates about the relative susceptibility of children compared with adults, we have performed sensitivity analyses with respect to this parameter, varying relative susceptibility from 40% to 70%. The attack ratio is highly sensitive to this parameter, as shown in figures 1–6*c,d*. We recall that individual susceptibility can be reduced by public health measures such as physical distancing and the usage of face masks, additional personal protective equipment and appropriate hygienic measures. Our investigations can therefore be used to provide readily available tables for decision-makers to investigate the impact of different public health measures to help inform the safe reopening of schools.

There are several limitations to the analysis presented here. We have assumed homogeneity among the two age classes which span large age groups: those individuals under 12 years and those individuals 12 years and above. The potential differences or heterogeneity in activity levels, susceptibility to infection by SARS-CoV-2 and vaccination coverage within each age group may be accounted for in subsequent studies. Furthermore, we have not accounted for variable vaccination rates: our study aims at providing an indication of the required levels of public health measures necessary to contain an outbreak within the children population, while assuming that all the parameters are ‘frozen’ at the current values. We believe this simplification can still give important insights, as the time variability of factors like vaccine uptake, individual behaviour and changes in transmissibility, possibly due to new emerging variants, is difficult to forecast in the context of COVID-19. Finally, while our work can give insights on the impact of several public health measures on the effective attack rate of children, we also remark that, in order to reduce the number of model parameters, we have assumed that the removal rate *γ_i_* (describing removal via natural recovery or isolation by public health measures) is the same for both symptomatic and asymptomatic individuals. In a pre-pandemic setting, this is equivalent to assuming that the recovery time is the same. While this assumption may not accurately capture the effect of interventions like isolation upon symptoms, we argue that the difference may not be substantial in the presence of measures like regular testing of individuals (independently of symptoms) or test-and-trace measures with isolation of close contacts (whether symptomatic or not). The sensitivity analyses presented here can therefore give valuable information about the impact of this parameter on the attack ratio, while reducing the number of free parameters in the system.

## Data Availability

All data used in this research are public and their sources are reported within. The simulation code has been deposited in Zenodo: https://doi.org/10.5281/zenodo.6360558 [47].

## Appendix A. Calculating the final size and attack ratios

Let *S_i_*_,∞_ be the final size of susceptible populations (*i* = 1, 2), namely, *S_i_*_,∞_ = *S_i_*_,∞_. Similarly, let *S_i_*_,0_ = *S_i_* (0). Integrating the equations for susceptible populations, we get

$$S_{i,\infty \;}=S_{i,0}e^{-\beta_{i}\left[\frac{C_{i1}}{N_{1}}({\hat{I}}_{1}+\theta {\;\hat{\;A}}_{1})+\frac{C_{i2}}{N_{2}}({\hat{I}}_{2}+\theta {\;\hat{\;A}}_{2})\right]}\;,$$

where $\;{\hat{\;A}}_{i}=\int_{0}^{\infty }A_{i}(t)\;dt,\;$ and ${\hat{I}}_{i}=\int_{0}^{\infty }I_{i}(t)\;dt$.

Using the equation for *A* and *I*, we get

$$\frac{d(e^{\gamma t}A_{i})}{dt}=\frac{1-\rho }{\rho }\;\frac{d(e^{\gamma t}I_{i})}{dt},$$

from which we obtain

$$\;{\hat{\;A}}_{i}=\frac{1-\rho }{\rho }{\hat{I}}_{i}.$$

Substituting this into the equation for *S_i_*_,∞_ yields

$$S_{i,\infty \;}=S_{i,0}e^{-\beta_{i}\left[\frac{C_{i1}}{N_{1}}\left(1+\frac{\theta (1-\rho )}{\rho }\right){\hat{I}}_{1}+\frac{C_{i2}}{N_{2}}\left(1+\frac{\theta (1-\rho )}{\rho }\right){\hat{I}}_{2}\right]}\;=S_{i,0}e^{-\beta_{i}\left(1+\frac{\theta (1-\rho )}{\rho }\right)\left[\frac{C_{i1}}{N_{1}}\;{\hat{I}}_{1}+\frac{C_{i2}}{N_{2}}{\hat{I}}_{2}\right]}.$$

Finally, we observe that

$${\left(S_{i}+E_{i}+I_{i}+A_{i}\right)}^{\prime }=-\gamma_{i}(I_{i}+A_{i}).$$

Therefore, we get

$$S_{i,\infty }-S_{i,0}=-\gamma_{i}({\hat{I}}_{i}+{\;\hat{\;A}}_{i})\;\;=-\gamma_{i}\left[1+\frac{1-\rho }{\rho }\right]{\hat{I}}_{i}=-\frac{\gamma_{i}}{\rho }{\hat{I}}_{i}\;$$

and

$$\frac{\gamma_{i}}{\rho }{\hat{I}}_{i}\;=S_{i,0}-S_{i,\infty }.\;$$

This leads to

$$\frac{S_{i,\infty }}{S_{i,0}}=e^{-\beta_{i}\left[1+\frac{\theta (1-\rho )}{\rho }\right]\left[\frac{C_{i1}\rho /N_{1}}{gamma_{1}}\;(S_{1,0}-S_{1,\infty })+\frac{C_{i2}\rho }{N_{2}\gamma_{2}}(S_{2,0}-S_{2,\infty })\right]}.$$

Using *S*_1_(0) = (1 − *p*_1_)*N*_1_, *S*_2_(0) = *N*_2_, we have

$$\frac{S_{i,\infty }}{S_{i,0}}=e^{-\beta_{i}\left[1+\frac{\theta (1-\rho )}{\rho )}\;\right]}\left[\frac{C_{i1}\rho (1-p)}{\gamma_{1}}\;\left(1-\frac{S_{1,\infty }}{S_{1,0}}\right)+\frac{C_{i2}\rho }{\gamma_{2}}\left(1-\frac{S_{2,\infty }}{S_{2,0}}\right)\right]\;.$$

If we substitute $x_{1}=S_{1,\infty }/S_{1,0},$and $x_{2}=S_{2,\infty }/S_{2,0},$ we have

$$x_{i}=e^{-\beta_{i}\left[1+\frac{\theta (1-\rho )}{\rho }\right]\left[\frac{C_{i1}\rho (1-p)}{\gamma_{1}}\;(1-x_{1})+\frac{C_{i2}\rho }{\gamma_{2}}(1-x_{2})\right]}.$$

The particular form of the equation for *x_i_* leads us to define the modified transmission rates $\beta_{1}^{*}=\beta_{1}\left[1+\frac{\theta (1-\rho )}{\rho }\right]$ and $\beta_{2}^{*}=\beta_{2}\left[1+\frac{\theta (1-\rho )}{\rho }\right]$ used in analysis contained in the main text.
